# In-Depth Genome Characterization and Pan-Genome Analysis of Strain KMM 296, a Producer of Highly Active Alkaline Phosphatase; Proposal for the Reclassification of *Cobetia litoralis* and *Cobetia pacifica* as the Later Heterotypic Synonyms of *Cobetia amphilecti* and *Cobetia marina*, and Emended Description of the Species *Cobetia amphilecti* and *Cobetia marina*

**DOI:** 10.3390/biom14020196

**Published:** 2024-02-06

**Authors:** Olga Nedashkovskaya, Larissa Balabanova, Nadezhda Otstavnykh, Natalia Zhukova, Ekaterina Detkova, Aleksandra Seitkalieva, Evgenia Bystritskaya, Yulia Noskova, Liudmila Tekutyeva, Marina Isaeva

**Affiliations:** 1 G.B. Elyakov Pacific Institute of Bioorganic Chemistry, Far Eastern Branch, Russian Academy of Sciences, Prospect 100 Let Vladivostoku 159, Vladivostok 690022, Russia; lbalabanova1@gmail.com (L.B.); chernysheva.nadezhda@gmail.com (N.O.); sasha0788@inbox.ru (A.S.); ep.bystritskaya@yandex.ru (E.B.); noskovaiulia@yandex.ru (Y.N.); 2 Institute of Biotechnology, Bioengineering and Food Systems, Advanced Engineering School, Far Eastern Federal University, Ajax Bay 10, Russky Island, Vladivostok 690922, Russia; tekuteva.la@dvfu.ru; 3 A.V. Zhirmunsky National Scientific Center of Marine Biology, Far Eastern Branch, Russian Academy of Sciences, Palchevskogo Street 17, Vladivostok 690041, Russia; nzhukova35@list.ru; 4 Winogradsky Institute of Microbiology, Russian Academy of Sciences, Federal Research Centre “Fundamentals of Biotechnology”, Leninsky Ave. 33, bld. 2, Moscow 119071, Russia; detkovkate@rambler.ru

**Keywords:** marine bacteria, *Cobetia amphilecti*, *Cobetia marina*, *Cobetia crustatorum*, phylogenomics, core genome, pan-genome analysis, taxonomy

## Abstract

A strictly aerobic, Gram-stain-negative, rod-shaped, and motile bacterium, designated strain KMM 296, isolated from the coelomic fluid of the mussel *Crenomytilus grayanus*, was investigated in detail due to its ability to produce a highly active alkaline phosphatase CmAP of the structural family PhoA. A previous taxonomic study allocated the strain to the species *Cobetia marina*, a member of the family *Halomonadaceae* of the class *Gammaproteobacteria*. However, 16S rRNA gene sequencing showed KMM 296’s relatedness to *Cobetia amphilecti* NRIC 0815^T^. The isolate grew with 0.5–19% NaCl at 4–42 °C and hydrolyzed Tweens 20 and 40 and L-tyrosine. The DNA G+C content was 62.5 mol%. The prevalent fatty acids were C_18:1_ ω7c, C_12:0_ 3-OH, C_18:1_ ω7c, C_12:0_, and C_17:0_ cyclo. The polar lipid profile was characterized by the presence of phosphatidylethanolamine, phosphatidylglycerol, phosphatidic acid, and also an unidentified aminolipid, phospholipid, and a few unidentified lipids. The major respiratory quinone was Q-8. According to phylogenomic and chemotaxonomic evidence, and the nearest neighbors, the strain KMM 296 represents a member of the species *C. amphilecti*. The genome-based analysis of *C. amphilecti* NRIC 0815^T^ and *C. litoralis* NRIC 0814^T^ showed their belonging to a single species. In addition, the high similarity between the *C. pacifica* NRIC 0813^T^ and *C. marina* LMG 2217^T^ genomes suggests their affiliation to one species. Based on the rules of priority, *C. litoralis* should be reclassified as a later heterotypic synonym of *C. amphilecti*, and *C. pacifica* is a later heterotypic synonym of *C. marina*. The emended descriptions of the species *C. amphilecti* and *C. marina* are also proposed.

## 1. Introduction

The genus *Cobetia* was created by Arahal et al. [1] to reclassify the species *Halomonas marina*. At the time of writing, the genus *Cobetia* comprises five validly published species names, including *C. marina* as the type species, *C. amphilecti*, *C. crustatorum*, *C. litoralis*, and *C. pacifica*, all isolated from different marine environments [1,2,3]. The genus accommodates Gram-stain-negative, aerobic, heterotrophic, halophilic, and rod-shaped bacteria, which can move by means of a single polar flagellum and/or two to seven lateral flagella [1,2,3]. Many strains of the genus *Cobetia* have been reported as a source of molecules and activities of biotechnological interest, and the genomic analysis of these strains has revealed their potential for the biosynthesis of biosurfactants, aromatic hydrocarbon degradation, inorganic carbon fixation, the synthesis and production of polyhydroxy-alkanoates, surface colonization, and alginate degradation [4,5,6,7,8]. Earlier, in the course of a survey of *Halomonas*-like bacteria inhabiting different areas of the Northwest Pacific, the strain KMM 296 was isolated from the coelomic fluid of the mussel *Crenomytilus grayanus*, collected from the Sea of Japan, and initially identified as a representative of the species *C. marina* (formerly *Deleya marina*) [9,10]. Later, the results of phylogenetic analysis based on the 16S rRNA gene sequence revealed the closest relationship of the strain KMM 296 to the strain *C. amphilecti* NRIC 0815^T^, with 100% sequence similarity. It should be noted that the genome of the strain KMM 296 (GenBank accession no. NZ_JQJA00000000.1) was sequenced [11] due to its ability to produce the highly active periplasmic alkaline phosphatase CmAP belonging to the PhoA alkaline phosphatase family [9,10,11,12,13,14]. Although the structural, biochemical, and catalytic properties of CmAP have been thoroughly studied, its exact physiological role still remains unknown due to the presence of several genes encoding for alkaline phosphatases with different structures in the KMM 296 genome [11]. In addition, CmAP was found to exhibit dephosphorylating activity towards bacterial lipopolysaccharides (LPS, endotoxins) similarly to other PhoA alkaline phosphatases from invertebrates and mammals, including humans [15,16,17,18,19]. Human intestinal alkaline phosphatase (IAP) provides an innate defense against endotoxins by altering the molecules to eliminate their pyrogenicity, resulting in an overall decrease in inflammatory processes. Thus, new data on the LPS-detoxifying activity of CmAP might lead to the development of a novel therapeutic approach for neutralizing the effects of bacterial endotoxins, such as Crohn’s disease, endotoxic shock, aging, etc. [17,18,19].

In the present study, we clarify the taxonomic position of the strain KMM 296 as a member of the *C. amphilecti* species and specify the description of the species *C. amphilecti* based on the results of phylogenomic analysis, phenotypic characterization, and experiments on the DNA-DNA hybridization between the strains KMM 296 and *C. amphilecti* NRIC 0815^T^. Since the 16S rRNA gene does not provide sufficient resolution to delineate *Cobetia* species [3], a huge number of published genomes of *Cobetia* strains cannot be affiliated to the existing species or have been wrongly designated. As a result, the precise identification of *Cobetia* strains other than *C. marina* and *C. crustatorum* strains remains a challenge, largely due to the lack of genome sequences for the type strains of *C. litoralis*, *C. pacifica*, and *C. amphilecti*. Therefore, in this work, we have presented data on the sequencing and genome analysis of the type strains NRIC 0814^T^, NRIC 0815^T^, and NRIC 0813^T^. A comparison of the genomic sequences and phenotypic characteristics suggests that the species *C. amphilecti* and *C. litoralis* belong to a single species. Moreover, based on the data obtained in this study, we propose the species *C. marina* and *C. pacifica* to be considered as the representatives of a single species. In accordance with the rules of priority, *C. litoralis* should be reclassified as a later heterotypic synonym of *C. amphilecti*, and *C. pacifica* is a later heterotypic synonym of *C. marina*. The emended descriptions of the species *C. amphilecti* and *C. marina* are also proposed.

## 2. Materials and Method

### 2.1. Strain Cultivation

Strain KMM 296 was obtained from the collection of marine microorganisms (KMM) at the G.B. Elyakov Pacific Institute of Bioorganic Chemistry FEB RAS (Vladivostok, Russia) and cultivated at 28 °C on marine agar (MA, Difco) and stored at −80 °C in marine broth (Difco) supplemented with 20% (*v*/*v*) glycerol. The type strains *C. amphilecti* NRIC 0815^T^ (KMM 1561^T^), *C. litoralis* NRIC 0814^T^ (KMM 3880^T^), *C. pacifica* NRIC 0813^T^ (KMM 3879^T^), and *C. marina* LMG 2217^T^ were kindly provided to us by NODAI Culture Collection Center (NRIC, Tokyo University of Agriculture, Tokyo, Japan) and the Belgian Coordinated Collection of Microorganisms (BCCM, Ghent University, Ghent, Belgium), respectively, and used as the reference strains for comparative taxonomic analysis.

### 2.2. Morphological, Biochemical, and Physiological Characterization

The physiological, morphological, and biochemical properties of strain KMM 296 were studied using the standard methods. The isolate was also examined in the API 20E, API 20NE, API 50 CH, API 32 ID GN, and API ZYM galleries (bioMérieux, Marcy l’Etoile, France) according to the manufacturer’s instructions, except that the galleries were incubated at 28 °C. Gram staining was performed as recommended in [20]. Oxidative or fermentative utilization of glucose was determined on Hugh and Leifson’s medium modified for marine bacteria [21]. Catalase activity was tested via addition of 3% (*v*/*v*) H_2_O_2_ solution to a bacterial colony and observation for the appearance of gas. Oxidase activity was determined by using tetramethyl-*p*-phenylenediamine. Degradation of agar, starch, casein, gelatin, chitin, DNA, and urea and production of acid from carbohydrates, hydrolysis of Tween 80, nitrate reduction, production of hydrogen sulfide, acetoin (Voges-Proskauer reaction), and indole were tested according to standard methods [20]. The temperature range for growth was assessed on marine agar (MA). Tolerance to NaCl was assessed in medium containing 5 g Bacto Peptone (Difco), 2 g Bacto Yeast Extract (Difco), 1 g glucose, 0.02 g KH_2_PO_4_, and 0.05 g MgSO_4_·7H_2_O per liter of distilled water with 0, 0.5, 1.0, 1.5, 2.0, 2.5, 3, 4, 5, 6, 8, 10, 12, 15, 17, 19, and 20% (*w*/*v*) of NaCl. Susceptibility to antibiotics was examined via the routine disc diffusion plate method. Discs were impregnated with the following antibiotics: ampicillin (10 μg), benzylpenicillin (10 U), carbenicillin (100 μg), cefalexin (30 μg), cefazolin (30 μg), chloramphenicol (30 μg), erythromycin (15 μg), gentamicin (10 μg), kanamycin (30 μg), lincomycin (15 μg), nalidixic acid (30 μg), neomycin (30 μg), ofloxacin (5 μg), oleandomycin (15 μg), oxacillin (10 μg), polymyxin B (300 U), rifampicin (5 μg), streptomycin (30 μg), tetracycline (5 μg), and vancomycin (30 μg).

### 2.3. Whole-Cell Fatty Acid, Polar Lipid, and Respiratory Quinone Composition

For comparative whole-cell fatty acid and polar lipid analysis, the strains KMM 296 and *C. amphilecti* NRIC 0815^T^ were grown under optimal physiological conditions for both strains at 30 °C for 24 h on MA. Cellular fatty acid methyl esters (FAMEs) were prepared according to the methods described by Sasser [22], using the standard protocol of Sherlock Microbial Identification System (version 6.0, MIDI), and analyzed using a GC-21A chromatograph (Shimadzu) equipped with a fused-silica capillary column (30 m × 0.25 mm) coated with Supercowax-10 and SPB-5 phases (Supelco) at 210 °C. FAMEs were identified by using equivalent chain-length measurements and comparing the retention times to those of authentic standards. The polar lipids of the strains studied were extracted using the chloroform/methanol extraction method of Bligh and Dyer [23]. Two-dimensional TLC of polar lipids was carried out on silica gel 60 F254 (10 × 10 cm; Merck) using chloroform/methanol/water (65:25:4, *v*/*v*) in the first dimension and chloroform/methanol/acetic acid/water (80:12:15:4, *v*/*v*) in the second dimension [24]. The spray reagent used to reveal the spots was molybdophosphoric acid. Isoprenoid quinones were extracted with chloroform/methanol (2:1, *v*/*v*) and purified via TLC using a mixture of *n*-hexane and diethyl ether (85:15, *v*/*v*) as the solvent. The isoprenoid quinone composition of the strain KMM 296 was characterized via HPLC (Shimadzu LC-10A) using a reversed-phase type Supelcosil LC-18 column (15 cm × 4.6 mm) and acetonitrile/2-propanol (65:35, *v*/*v*) as a mobile phase at a flow rate of 0.5 mL min^−1^, as described previously [25].

### 2.4. The 16S rRNA Gene Sequencing and DNA–DNA Hybridization

DNA was extracted from 0.1–0.2 g of the bacterial cells (wet weight), using an extraction protocol by Sambrook and Russell [26]. PCR was carried out using the universal oligonucleotide primers 11F (5′-GTTTGATCMTGGCTCAG-3′) and 1492R (5′-TACGGYTACCTTGTTACGACTT-3′), as described by Weisburg [27], and the GeneAmp PCR System 2720 (Applied Biosystems, Singapore, Singapore). PCR amplicons were used as templates for sequencing amplification using a BigDye Terminator version 3.1 Cycle sequencing kit (Applied Biosystems). The purified sequencing products were analyzed via electrophoresis using a 50 cm capillary array with an ABI Prism 3130xL DNA sequencer (Applied Biosystems, Hitachi, Japan), and the sequence was assembled with SeqScape version 2.6 (Applied Biosystems). The sequences obtained were deposited in NCBI GenBank under the accession numbers presented by Noskova et al. [28] and analyzed against the referent phylotypes, based on the type strain 16S rRNA gene sequences and whole-genome assemblies in the EzBioCloud database [29].

The DNA–DNA hybridization between the strain KMM296 and *C. amphilecti* NRIC 0815^T^ was performed spectrophotometrically, and initial renaturation rates were recorded as described by De Ley et al. [30].

### 2.5. Whole-Genome Shotgun Sequencing and Phylogenetic Analysis

The genomic DNA was obtained from the bacterial cultures of eight *Cobetia* strains, namely, *C. amphilecti* NRIC 0815^T^, *C. litoralis* NRIC 0814^T^, *C. pacifica* NRIC 0813^T^, *Cobetia* sp. 1AS1, *Cobetia* sp. 2AS, *Cobetia* sp. 3AK, *Cobetia* sp. 10Alg 146, and *Cobetia* sp. 29-18-1, using NucleoSpin Microbial DNA Mini kit (Macherey-Nagel, Düren, Germany), following the manufacturer’s instructions. Whole-genome shotgun sequencing was carried out on an Illumina MiSeq platform using Nextera DNA Flex kits, with a 150 bp paired-end sequencing kit (Illumina, San Diego, CA, USA). The sequence quality was assessed via FastQC version 0.11.8 [FastQC. Available online: http://www.bioinformatics.babraham.ac.uk/projects/fastqc/ (accessed on 28 December 2021)], and reads were trimmed using Trimmomatic version 0.38 [31]. Filtered reads were assembled de novo with SPAdes version 3.15.3 [32]. The draft genomes of the strains *C. amphilecti* NRIC 0815^T^, *C. litoralis* NRIC 0814^T^, *C. pacifica* NRIC 0813^T^, *Cobetia* sp. 1AS1, *Cobetia* sp. 2AS, *Cobetia* sp. 3AK, *Cobetia* sp. 10Alg 146, and *Cobetia* sp. 29-18-1 were annotated using NCBI Prokaryotic Genome Annotation Pipeline (PGAP) [33] and deposited in GenBank/EMBL/DDBJ under the accession numbers JASCSA000000000, JARWKV000000000, JASCSB000000000, JARWKU000000000, JARWKQ000000000, JARWKR000000000, JARWKT000000000, and JARWKS000000000, respectively.

All publicly available *Cobetia* genomes were retrieved from the Reference sequence (RefSeq) database at NCBI using the NCBI-genome-download version 0.3.0 (https://github.com/kblin/ncbi-genome-download, accessed on 16 March 2023, *n* = 28) [34]. The accession numbers for the genomes used in this study are listed in Table 1.

The pan-genome for these 36 *Cobetia* strains (Table 1) was reconstructed using the microbial pan-genomics workflow in Anvi’o version 7.1 (minbit = 0.5; mcl-inflation = 2; min-occurrence = 1) [35]. The genomes were organized based on the distribution of gene clusters using the MCL algorithm (Distance: Euclidean; linkage: Ward). For average nucleotide identity (ANIm) calculation, we used the program ‘anvi-compute-genome-similarity’ with ‘-program pyANI’ flag. Amino acid identity (AAI) and in silico DNA–DNA hybridization (dDDH) values between the strains were calculated with the online server ANI/AAI-Matrix [36] and the TYGS platform (formula d4), respectively [37]. To produce the phylogenetic tree of the genus *Cobetia*, 1432 single-copy core gene sequences for each strain were extracted from the pan-genome and concatenated (composite length of 465,701 bp) using the program ‘anvi-get-sequences-for-gene-clusters’ with ‘-concatenate-gene-clusters’ flag. Resulting FASTA files were cleaned up by removing nucleotide positions that had gap characters in more than 50% of the sequences using the trimAl version 1.4.1 [38]. A core-genome phylogeny was reconstructed with IQ-TREE version 2.2.0.3 under the WAG model with non-parametric bootstrapping using 100 replicates [39]. The pan-genome and core-genome modelling were estimated with PanGP v.1.0.1 using a power-law regression model based on Heap’s law and exponential regression, respectively, as described by Tettelin et al. [40].

Fonts and sizes in all figures were edited manually in Adobe Photoshop CC 2018 for better visualization.

## 3. Results and Discussion

### 3.1. Morphological, Biochemical, and Physiological Characterization

The strain KMM 296 was shown to be a strictly aerobic, heterotrophic, Gram-stain-negative and motile bacterium that formed a slightly yellow-colored colony on MA and required NaCl or seawater for growth. It was positive for cytochrome oxidase and catalase and did not hydrolyze agar, casein, gelatin, starch, Tween 80, DNA, urea, or chitin (Table 2).

The strains KMM 296 and *C. amphilecti* NRIC 0815^T^ shared many common phenotypic features, such as the respiratory type of metabolism, motility by means of 1–2 polar and/or 2–3 lateral flagella, the ability to grow at 4–42 °C, the presence of catalase, alkaline phosphatase, esterase (C4), esterase lipase (C8), leucine arylamidase, acid phosphatase, and α-glucosidase activities, and the assimilation of sucrose, maltose, sodium malonate, glycogen, D-mannitol, D-glucose, 3-hydroxybutyric acid, and L-proline (Table 2). They could not synthesize arginine dihydrolase, lipase (C14), cystine arylamidase, α-chymotrypsin, N-acetyl-β-glucosaminidase, β-glucosidase, α-galactosidase, β-glucuronidase, α-mannosidase, or α-fucosidase; hydrolyze agar, chitin, aesculin, gelatin, starch, urea, or Tween 80; produce acid from D-mannose, melibiose, raffinose, L-rhamnose, D-ribose, N-acetylglucosamine, inositol, D-sorbitol, glycerol, or D-mannitol; reduce nitrate to nitrite or assimilate L-arabinose, D-mannose, N-acetylglucosamine, adipate, phenylacetate, itaconic acid, sodium acetate, propionic acid, trisodium citrate, or 4-hydroxybenzoic acid. However, the strain KMM 296 can be distinguished from its closest phylogenetic relative by several phenotypic traits, including the presence of cytochrome oxidase and the ability to assimilate capric and valeric acids, the inability to produce acid from a set of carbohydrates and to assimilate D-glucose, D-mannitol, maltose, D-gluconate, L-malate, L-rhamnose, N-acetylglucosamine, D-ribose, inositol, suberic acid, lactic acid, L-alanine, potassium 5-ketogluconate, 3-hydroxybenzoic acid, L-serine, salicin, melibiose, L-fucose, L-arabinose, L-histidine, and potassium 2-ketogluconate (Table 2).

The above findings can extend the phenotypic characteristics that were reported for the species *C. amphilecti* by Romanenko et al. [3] after the justification of the placement of the strain KMM 296 in this species.

### 3.2. Whole-Cell Fatty Acid, Polar Lipid, and Respiratory Quinone Composition in the Strains KMM 296 and C. amphilecti NRIC 0815^T^

The predominant fatty acids (>5% of the total) in the strain KMM 296 were C_18:1_ ω7*c*, C_12:0_ 3-OH, C_18:1_ ω7*c*, C_12:0_, and C_17:0_ cyclo (Table 3).

**Figure 1:**
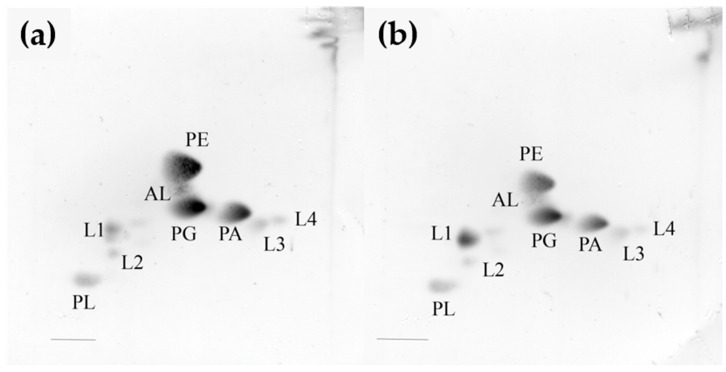
Two-dimensional thin-layer chromatogram of polar lipids extracted from the strains KMM 296 (**a**) and *C. amphilecti* NRIC 0815^T^ (**b**). PE, phosphatidylethanolamine; PG, phosphatidylglycerol; PC, phosphatidylcholine; PA, phosphatidic acid; AL, unidentified amino lipid; L, unidentified lipid; PL, unknown phospholipid.

The polar lipid profile of the strain KMM 296 was characterized by the presence of phosphatidylethanolamine, phosphatidylglycerol, phosphatidic acid, an unidentified aminolipid, an unidentified phospholipid, and unidentified lipids, and it was found to be similar to that of *C. amphilecti* NRIC 0815^T^ (Figure 1a,b). The major respiratory quinone was Q-8, which is common among members of the class *Gammaproteobacteria*.

### 3.3. The 16S rRNA Gene Phylogenetic Analysis of the Strains KMM 296, C. marina LMG 2217^T^, and C. amphilecti NRIC 0815^T^

Phylogenetic analysis based on 16S rRNA gene sequences revealed that the strain KMM 296 demonstrated only 99.5% sequence similarity to *C. marina* LMG 2217^T^ (=JCM 21022^T^), whereas it was found to be identical to the type strain of another validly published *Cobetia* species, *C. amphilecti*, with 100% sequence similarity [3,28]. This suggests that the strain KMM 296 can be placed in this species instead of *C. marina*, the strains of which were predominantly isolated from degraded alga thallus [10]. In addition, the comparative genome analysis and phylogenomic analysis of the family *Halomonadaceae*, implemented by Tang et al. [41], indicated that the significant differences between *C. marina* JCM 21022^T^ and the strain KMM 296 (formerly *C. marina* KMM 296) resulted from sequence insertions or deletions and chromosomal recombination [13,41].

### 3.4. GC Comparison between Strains KMM 296 and C. amphilecti NRIC 0815^T^

The genomic GC content of the strain KMM 296 was 62.5 mol%, as determined using the genome sequencing data [13]. This value was slightly lower than that obtained via the thermal denaturation method (62.7 mol%) [10]. The DNA–DNA relatedness between the isolate KMM 296 and the strain *C. amphilecti* NRIC 0815^T^, which was determined via the experimental DNA–DNA hybridization method, was 92%. This value was higher than the 70% threshold used for assigning bacterial strains to the same genomic species [42] and it strongly suggested that the two strains belong to the single species, *C. amphilecti.*

The closest evolutionary distances between the type strains of the species *C. amphilecti* and *C. litoralis*, on the one hand (99.93% 16S rRNA gene sequence similarity), and *C. marina* and *C. pacifica*, on the other hand (100% 16S rRNA gene sequence similarity), calculated using the EzBioCloud 16S RNA database tools and discussed earlier [28], also suggest that the species *C. amphilecti* and *C. litoralis* belong to one species and the species *C. marina* and *C. pacifica* could also be joined to a single species.

However, the comparison of the whole-genome sequences of all *Cobetia* spp. strains, which are currently deposited in the NCBI GenBank database (Table 1), revealed that each genome contains up to seven 16S rRNA genes, with different levels of similarity (99.86–100%) within one strain, as well as between the different species [28]. Therefore, comprehensive whole-genome-based analyses are required for *Cobetia* species demarcation.

### 3.5. Whole-Genome-Based Phylogeny and Analysis of Cobetia Strains

In total, 36 *Cobetia* strains were chosen for phylogenetic and comparative analyses, 8 of which have been sequenced in this study (3 type strains, NRIC 0814^T^, NRIC 0815^T^, and NRIC 0813^T^, and 5 isolates, 2AS1, 2AS, 1AS1, 29-18-1, and 10Alg 146). Twenty-eight genomes of the *Cobetia* spp. isolates were retrieved from the RefSeq database at NCBI. The genomic dataset included the genomes of the type strains of five *Cobetia* species according to the previous taxonomy classification [1,2,3], nine *Cobetia* spp. strains, and twenty-two unclassified *Cobetia* isolates. The overall features of the genomes are listed in Table 1. The genome size ranged from 3.1 to 4.6 Mbp, while the GC content varied slightly and was 62–63% except for four strains with 57–57.5%, including *C. crustatorum* JO1^T^ (Table 1). Apparently, such values might be due to the difference in the genome completeness levels. According to the NCBI Quality analysis (CheckM) [43], the assemblies showed 81.26–99.65% completeness and 0.49–2.93% contamination (Table 1).

**Figure 2:**
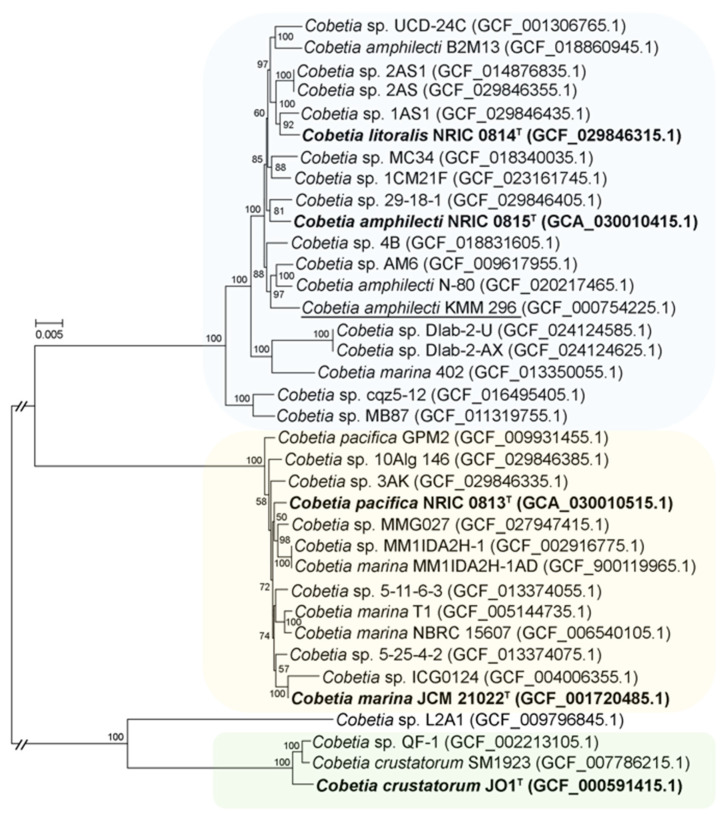
Maximum-likelihood phylogeny of the genus *Cobetia* based on concatenated 1432 single-copy core gene sequences and reconstructed with IQ-TREE with non-parametric bootstrapping using 100 replicates, including Bar, with 0.005 substitutions per amino acid position. The corresponding accession numbers for the genomes are given in parentheses. The type strains of the previously classified *Cobetia* species are shown in bold; strain KMM 296 is underlined.

A core-genome phylogeny was used to estimate the phylogenetic relationships of the *Cobetia* strains (Figure 2). According to the phylogenetic tree, the strains fall into four clades with subclades (bootstrap values = 100).

The first one included three subclades and contained nineteen strains, among which two type strains, *C. litoralis* NRIC 0814^T^ and *C. amphilecti* NRIC 0815^T^, clustered at the same subclade. The second clade consisted of 13 strains, including the type strains *C. pacifica* NRIC 0813^T^ and *C. marina* JCM 21022^T^. The third clade branched distantly and consisted of two *C. crustatorum* and two *Cobetia* spp. strains. According to the obtained topology of the phylogenomic tree, it is clear that five described species may actually represent only three species (Figure 2).

The values of the phylogenomic metrics ANIb, AAI, and dDDH are further evidence redefining the species assignments within the genus *Cobetia* (Table S1). The obtained ANIb, AAI, and dDDH values for fourteen strains showing the same phylogenomic grouping, including the type strains *C. litoralis* NRIC 0814^T^ and *C. amphilecti* NRIC 0815^T^ (Figure 2), were found to be 96.43–96.95%, 97.64–99.99%, and 70.1–100%, respectively. The group of thirteen strains clustered together, including the type strains *C. pacifica* NRIC 0813^T^ and *C. marina* JCM 21022^T^ (Figure 2), showed the ranges of 97.14–98.19%, 98.2–99.99%, and 80.4–100% for the ANIb, AAI, and dDDH values, respectively. These values between *C. crustatorum* JO1^T^, *C. crustatorum* SM1923, and *Cobetia* sp. QF-1 (Figure 2) were 98.4–98.9%, 98.44–99.18%, and 89.5–91.7%, respectively. The strain *Cobetia* sp. L2A1 shared corresponding values of 80.8–85.7%, 86.27–90.89%, and 24.4–29.3% with the *Cobetia* spp. type strains. Considering the thresholds of 95–96% ANI, 95–96% AAI, and 70% dDDH defined for species demarcation, the type strains *C. litoralis* NRIC 0814^T^, *C. amphilecti* NRIC 0815^T^, *C. pacifica* NRIC 0813^T^, and *C. marina* JCM 21022^T^ do not belong to the corresponding originally assigned species [44]. The high values confirm the phylogenetic grouping of those strains, which are likely to represent two instead of four separate species (Figure 2). The phylogenomic metrics of the strain *Cobetia* sp. L2A1 were below the cutoff scores, implying that it might belong to a novel species of the genus *Cobetia* (Figure 2, Table S1).

### 3.6. Pan-Genome-Based Phylogeny and Analysis of Cobetia Strains

**Figure 3:**
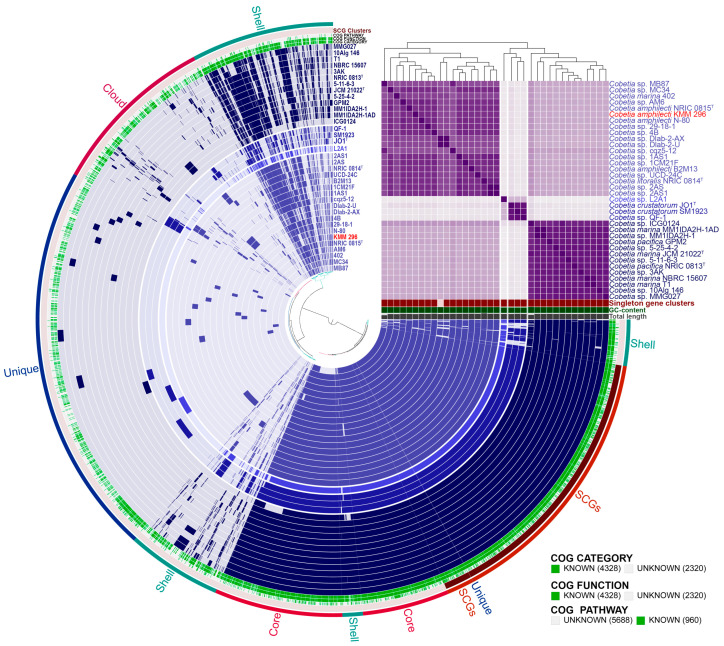
The pan-genome of 36 *Cobetia* spp. strains. Circle bars represent the presence/absence of 6648 pan-genomic clusters in each genome. Gene clusters are organized as core, soft-core, shell, and cloud gene clusters using Euclidian distance and Ward ordination. The heatmap in the upper right corner displays pairwise values of average nucleotide identity (ANIm) in percentages. The strain KMM 296 is colored in red.

The pan-genome analysis of the genus *Cobetia* was performed to determine its genetic heterogeneity and phylogenetic relationships (Figure 3). The pan-genome is presented by a set of gene clusters (GCs), among which are the conserved core and the accessory shell and the cloud genes. The core genes are found in ≥95% of the genomes, the shell genes are found in more than 10% and less than 95% of the genomes, and the cloud genes are present in ≤10% of the genomes. Moreover, the single-copy genes (SCGs), as a part of the core, are found in all strains, while the unique genes (singletons) from the cloud are strain-specific. The pan-genome of 36 strains of the genus *Cobetia* comprised a total of 6648 gene clusters (distance: Euclidean; linkage: Ward) with 123,892 gene calls that include 2471 core gene clusters (93,289 genes in all 36 genomes), 1469 gene clusters in the shell (26,722 genes), and 2708 in the cloud (3881 genes), including 1902 gene clusters (1920 genes) of singletons. It is interesting that 62 GCs were found belonging exclusively to the strains grouped with the type strain *C. crustatorum* JO1^T^, while 20 GCs were found exclusively in the strains, clustered with the type strains *C. pacifica* NRIC 0813^T^ and *C. marina* JCM 21022^T^ (Figure 3). However, only one GC was found to be common for the 14 strains, grouped with *C. litoralis* NRIC 0814^T^ and *C. amphilecti* NRIC 0815^T^, indicating a high rate of genomic intraspecies reorganization within their populations and the species diversification depending on their free or host-associated lifestyle [28].

The core and unique gene clusters were further annotated into COG classes. The core genes were related to the following classes: translation and ribosomal biogenesis (10.48%), amino acid transport and metabolism (9.58%), cell envelope synthesis (7.35%), energy production and conversion (6.95%), transcription (6.65%), carbohydrate metabolism and transport (6.35%), lipid metabolism (6.06%), inorganic ion transport and metabolism (5.91%), coenzyme metabolism (5.46%), post translational modifications (5.21%), replication and repair (4.42%), and signal transduction (3.82%). The classes for nucleotide metabolism and transport, secondary metabolite synthesis, defense mechanisms, cell cycle control, and intracellular trafficking and secretion were in a minority in the core (1.24–3.07%).

**Figure 4:**
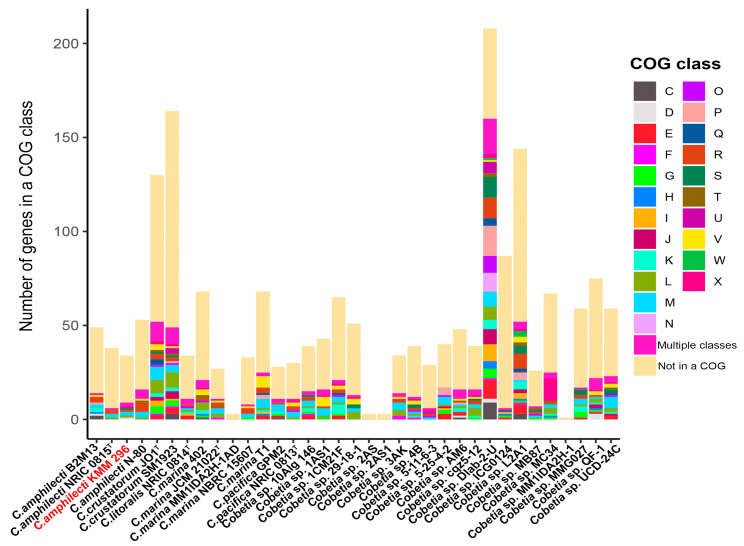
The number of unique genes assigned to a functional class (COG) among strains of *Cobetia* spp. Classes: C, energy production and conversion; D, cell cycle control and mitosis; E, amino acid metabolism and transport; F, nucleotide metabolism and transport; G, carbohydrate metabolism and transport; H, coenzyme metabolism, I, lipid metabolism; J, translation; K, transcription; L, replication and repair; M, cell wall/membrane/envelope biogenesis; N, cell motility; O, post-translational modification, protein turnover, chaperone functions; P, inorganic ion transport and metabolism; Q, secondary structure; R, general functional prediction only; S, function unknown; T, signal transduction; U, intracellular trafficking and secretion; V, defense mechanisms; W, extracellular structures; X, mobilome: prophages, transposons. Multiple classes—genes assigned to two or more COG categories. Not in a COG—COG not defined. The strain KMM 296 is colored in red.

It is worth noting that each of the *Cobetia* genomes contains from one to two hundred and eight unique genes (Figure 4). The largest numbers of singletons were observed in the genomes of *Cobetia* sp. Dlab-2-U (208 genes), *C*. *crustatorum* SM1923 (164), *Cobetia* sp. L2A1 (144), and *Cobetia* sp. QF-1 (130). The genomes of *C. marina* MM1IDA2H-1AD, *Cobetia* sp. 2AS, *Cobetia* sp. 2AS1, and *Cobetia* sp. MM1IDA2H-1 account for the smallest number of the unique genes—3, 3, 3, and 1, respectively. The remaining genomes harbor from 26 to 87 unique genes. According to the COG class annotation of these unique genes, the most abundant functional classes were cell wall/membrane/envelope biogenesis (14.12% of total unique gene clusters), general functional prediction only (10.63%), replication and repair (9.8%), defense mechanisms (6.98%), amino acid metabolism and transport (6.48%), and transcription (6.15%).

**Figure 5:**
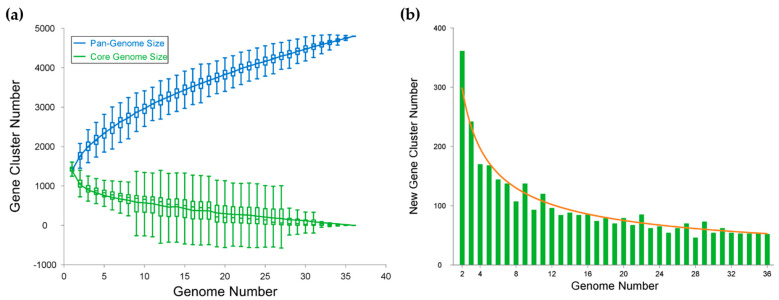
Pan-genome modelling. (**a**) Gene accumulation curves for the pan-genome and the core genome of 36 *Cobetia* genomes. Pan-genome curve: *y* = 889.06*x*^0.43^ + 535.51. Core genome curve: *y* = 1250.45e^−0.07*x*^ − 29.77. (**b**) The new gene cluster number plot, curve: *y* = 452.537*x*^−0.6^.

According to the modeling of the pan- and core genome sizes upon the addition of new genomes into the dataset, the pan-genome of *Cobetia* spp. is an open one with a γ value of 0.43 (Figure 5a). The best-fit regression curve for a pan-genome is rising upwards, implying an expanding pan-genome, while the core genome’s curve tends to reach a plateau [40]. Moreover, the fitting of the curve to a power law showed that the number of the new gene cluster discovery with the adding of the new genome would add 52 and 29 genes to the pan-genome, as predicted for the 37th and 100th sequenced genomes, respectively (Figure 5b).

## 4. Conclusions

The genus *Cobetia* currently includes five species with validly described names, *C. marina*, *C. amphilecti*, *C. crustatorum*, *C. litoralis*, and *C. pacifica*. However, the shared 16S rRNA gene sequence identity being almost 100% between the species *C. amphilecti* and *C. litorali*, as well as between the species *C. marina* and *C. pacifica*, does not allow us to delineate the *Cobetia* species. In this work, we have presented data on the sequencing and genome analysis of the type strains of *C. amphilecti, C. litoralis,* and *C. pacifica*. Our phylogenomic and pan-genomic analyses of the genus *Cobetia*, based on the 8 genome sequences presented in this study and 28 publicly available genome sequences, confirm the taxonomic status of only three species: *C. marina*, *C. amphilecti*, and *C. crustatorum*. The strain *Cobetia* sp. L2A1 was proven to be a member of a novel species. In addition, the taxonomic status of all *Cobetia* strains with available genomes has been clarified.

In summary, based on the results of genomic, phylogenetic, phenotypic, and chemotaxonomic studies, we suggested that the species *C. litoralis* should be placed in the species *C. amphilecti*, and the species *C. pacifica* should be included in the species *C. marina*. In accordance with the priority rules, *C. litoralis* should be reclassified as a later heterotypic synonym of *C. amphilecti* and *C. pacifica* is a later heterotypic synonym of *C. marina*. The emended descriptions of the species *C. amphilecti* and *C. marina* are proposed.

**Emended description of the species *Cobetia amphilecti* (Romanenko et al., 2013)**

The description of the species *Cobetia amphilecti* and *Cobetia litoralis* is as given by Romanenko et al. (2013), with the following amendments. Cells are oxidase-positive and motile by means of 1–2 polar and/or 2–5 lateral flagella. Some strains can request seawater or NaCl for growth. The predominant fatty acids (>5% of the total fatty acids) are C_16:1_ ω7*c*, C_12:0_ 3-OH, C_16:0,_ C_18:1_ ω7*c*, C_17:0_ cyclo, and C_12:0_. The polar lipid profile is characterized by the presence of phosphatidylethanolamine, phosphatidylglycerol, phosphatidic acid, an unidentified aminolipid, the two unidentified phospholipids, and the four unidentified lipids. The major respiratory quinone is Q-8. The genomic DNA G+C content is 62.5 mol%.

**Emended description of the species *Cobetia marina* (Cobet et al., 1970, Arahal et al., 2002, Romanenko et al., 2013)**

The description of the species *Cobetia marina* and *Cobetia pacifica* is as given by Arahal et al. (2002) and Romanenko et al. (2013), with the following amendments. Cells are oxidase-positive and motile by means of 1–2 polar and/or 2–5 lateral flagella. The predominant fatty acids (>5% of the total fatty acids) were C_16:1_ ω7*c*, C_12:0_ 3-OH, C_16:0,_ C_17:0_ cyclo, C_18:1_ ω7*c*, and C_12:0_. The polar lipid profile is characterized by the presence of phosphatidylethanolamine, phosphatidylglycerol, phosphatidic acid, an unidentified aminolipid, the two unidentified phospholipids, and the four unidentified lipids. The major respiratory quinone is Q-8. The genomic DNA G+C content is 62.2–62.4 mol%.

## Figures and Tables

**Table 1:** The accession numbers and general attributes of 36 *Cobetia* spp. genomes used in this study.

Strain	Accession ID	Genome Size, bp	G+C (mol%)	Contigs	Completeness (%)	Contamination (%)	Isolation Source
*Cobetia* sp. UCD-24C	GCF_001306765.1	4,229,986	62.5	51	98.9	1.76	Seagrass sediment
*C. amphilecti* B2M13	GCF_018860945.1	4,289,324	62.5	58	95.88	0.91	Artificial alginate particle
*Cobetia* sp. 2AS1	GCF_014876835.1	4,248,424	62.5	49	95.96	0.9	Coastal sediment
*Cobetia* sp. 2AS	GCF_029846355.1	4,247,060	62.5	39	99.15	1.3	
*Cobetia* sp. 1AS1	GCF_029846435.1	4,235,090	62.5	43	99.27	1.71	Coastal seawater
***C. litoralis* NRIC 0814^T^**	GCF_029846315.1	4,621,254	62.5	51	99.15	2.93	Sandy sediment
*Cobetia* sp. MC34	GCF_018340035.1	4,022,416	62.5	175	95.47	0.87	Fish-landing facility
*Cobetia* sp. 1CM21F	GCF_023161745.1	4,261,659	62.5	24	95.88	0.49	Sea cave
*Cobetia* sp. 29-18-1	GCF_029846405.1	4,117,019	62.5	70	99.35	1.48	*Esperiopsis digitata*
***C. amphilecti* NRIC 0815^T^**	GCA_030010415.1	4,171,304	62.5	112	98.54	1.34	Internal tissue, *Amphilectus digitatus*
*Cobetia* sp. 4B	GCF_018831605.1	4,325,922	62.5	3	99.41	1.33	Current Humbolt system, *Heterostera chilensis*
*Cobetia* sp. AM6	GCF_009617955.1	4,229,996	62.5	1	98.74	1.36	Japan: Tokyo
*C. amphilecti* N-80	GCF_020217465.1	4,160,095	62.5	1	98.74	1.28	Marine sediment
*C. amphilecti* KMM 296	GCF_000754225.1	3,965,007	62.5	97	98.29	1.28	*Crenomytilus grayanus*
*Cobetia* sp. Dlab-2-U	GCF_024124585.1	4,144,083	62.5	137	96.63	1.3	Coral surface mucus layer and tissue *Diploria labyrinthiformis*
*Cobetia* sp. Dlab-2-AX	GCF_024124625.1	4,001,795	62.5	20	96.29	1.3	
*C. marina* 402	GCF_013350055.1	3,978,956	62.5	132	95.93	0.6	Seawater, aquarium
*Cobetia* sp. cqz5-12	GCF_016495405.1	4,209,007	62.5	1	99.45	1.71	Brown algae *Sargassum fusiforme*
*Cobetia* sp. MB87	GCF_011319755.1	3,101,384	62.5	12	81.26	1.58	Sea cucumber gut
*C. pacifica* GPM2	GCF_009931455.1	4,195,186	62.5	1	99.59	1.33	*Neopyropia tenera*
*Cobetia* sp. 10Alg 146	GCF_029846385.1	4,095,141	62.5	33	99.44	1.68	*Ahnfeltia tobuchiensis*
*Cobetia* sp. 3AK	GCF_029846335.1	4,073,243	62.5	58	99.44	1.71	Coastal seawater
***C. pacifica* NRIC 0813^T^**	GCA_030010515.1	4,066,371	62.5	42	99.44	1.3	Sandy sediment
*Cobetia* sp. MMG027	GCF_027947415.1	4,168,882	62.5	47	99.24	1.71	-
*Cobetia* sp. MM1IDA2H-1	GCF_002916775.1	4,151,052	62	88	99.65	1.33	Eulittoral intertidal pond at sea level
*C. marina* MM1IDA2H-1AD	GCF_900119965.1	4,155,178	62	105	99.65	1.33	-
*Cobetia* sp. 5-11-6-3	GCF_013374055.1	4,120,053	62.5	40	99.44	1.3	Seaweed
*C. marina* T1	GCF_005144735.1	4,177,239	62	21	99.44	1.28	Saltwater
*C. marina* NBRC 15607	GCF_006540105.1	4,184,377	62.5	113	99.24	1.68	-
*Cobetia* sp. 5-25-4-2	GCF_013374075.1	4,118,344	62.5	41	99.44	1.3	Seaweed
*Cobetia* sp. ICG0124	GCF_004006355.1	3,345,506	63	1	90.85	2.1	Marine waters
***C. marina* JCM 21022^T^**	GCF_001720485.1	4,176,400	62.5	1	99.51	1.51	Littoral water
*Cobetia* sp. L2A1	GCF_009796845.1	4,118,938	57.5	1	98.9	1.28	Arctic Ocean beach
*Cobetia* sp. QF-1	GCF_002213105.1	4,084,184	57.5	31	99.04	0.93	Seawater
*C. crustatorum* SM1923	GCF_007786215.1	4,215,468	57.5	163	98.83	0.82	Surface seawater
***C. crustatorum* JO1^T^**	GCF_000591415.1	4,049,952	57.5	138	90.05	0.52	Fermented shrimp

Type strains, *C. litoralis* NRIC 0814^T^, *C. amphilecti* NRIC 0815^T^, *C. pacifica* NRIC 0813^T^, *C. marina* JCM 21022^T^, *C. crustatorum* JO1^T^, are shown in bold.

**Table 2:** The different characteristics of the strain KMM 296 and its closest relative *C. amphilecti* NRIC 0815^T^.

Characteristic	KMM 296	*C. amphilecti* NRIC 0815^T^
Source and site of isolation	Mollusk *C. grayanus*, the Sea of Japan, Pacific Ocean	Sponge *A. digitatus*, the Gulf of Alaska, Pacific Ocean
Temperature range for growth (°C)	4–42	4–42
Salinity range for growth (% NaCl)	0.5–19	0–20
Nitrate reduction	-	+
Hydrolysis of:		
Tween 80	-	+
DNA	-	+
Acid production from:		
D-Fructose, D-lactose	-	+
L-Arabinose, D-melibiose, L-rhamnose	+	-
Assimilation of:		
Amygdalin, maltose, sodium malonate, glycogen, capric acid, valeric acid, 3-hydroxybutiric acid, L-proline	+	-
N-acetylglucosamine, L-serine	-	+
Enzyme activities:		
Valine arylamidase, cysteine arylamidase	-	+
Trypsin	+	-
DNA G+C content (mol%)	62.5	62.5

Both strains were positive in the following tests: respiratory type of metabolism and motility; slightly yellowish colony color; hydrolysis of tyrosine and Tweens 20 and 40; catalase, oxidase, alkaline phosphatase, esterase (C4), esterase lipase (C8), leucine arylamidase, acid phosphatase, naphthol-AS-BI-phosphohydrolase, and α-glucosidase activities via the PNPG test; acid production from D-galactose, D-glucose, and D-mannose; assimilation of L-arabinose, D-melibiose, L-rhamnose, sucrose, maltose, and D-mannitol; susceptibility to ampicillin, carbenicillin, cephalexin, cephazolin, chloramphenicol, erythromycin, gentamicin, kanamycin, nalidixic acid, neomycin, ofloxacin, polymyxin, rifampicin, streptomycin, tetracycline, and vancomycin; and resistance to benzylpenicillin, lincomycin, oleandomycin, and oxacillin. Both strains were negative for the following tests: arginine dihydrolase, lipase (C14), α-chymotrypsin, N-acetyl-β-glucosaminidase, α-galactosidase, β-galactosidase, β-glucosidase, β-glucuronidase, α-mannosidase, and α-fucosidase activities; the hydrolysis of aesculin, agar, casein, chitin, gelatin, starch, and urea; acid production from raffinose, D-ribose, D-xylose, sucrose, trehalose, D-cellobiose, N-acetylglucosamine, glycerol, inositol, D-sorbitol, sodium acetate, trisodium citrate, and D-mannitol; and the production of H_2_S and indole.

**Table 3:** Fatty acid profiles (%) of the strains KMM 296 and *C. amphilecti* NRIC 0815^T^.

Fatty Acid *	KMM 296	*C. amphilecti* NRIC 0815^T^
C10:0	3.2	4.3
C12:0	11.0	8.7
C16:0	21.6	21.9
C17:0 cyclo	5.2	9.4
C16:1 ω7c	38.2	32.5
C18:1 ω7c	14.9	12.7
C19:1 ω6c	1.6	tr
C12:0 3-OH	15.8	20.3

* Data are from the present study. tr, trace amount (≤1%).

## Data Availability

The whole-genome shotgun sequences of the strains *Cobetia amphilecti* NRIC 0815^T^, *Cobetia litoralis* NRIC 0814^T^, *Cobetia pacifica* NRIC 0813^T^, *Cobetia* sp. 1AS1, *Cobetia* sp. 2AS, *Cobetia* sp. 3AK, *Cobetia* sp. 10Alg 146, and *Cobetia* sp. 29-18-1 were deposited in GenBank/EMBL/DDBJ under the accession numbers JASCSA000000000, JARWKV000000000, JASCSB000000000, JARWKU000000000, JARWKQ000000000, JARWKR000000000, JARWKT000000000, and JARWKS000000000, respectively.

## Supplementary Materials

The following supporting information can be downloaded at: https://www.mdpi.com/article/10.3390/biom14020196/s1, Table S1: The phylogenomic metrics ANIb, AAI, and dDDH calculated for the *Cobetia* spp. strains and isolates.
